# Effects of eye movement exercise combined with conventional physical therapy on chronic nonspecific neck pain: a randomized controlled trial protocol

**DOI:** 10.3389/fspor.2026.1929676

**Published:** 2026-10-14

**Authors:** Li Gou, Shiya Yu, Zhuo Wang, Yuan Kang, Yue Hou, Lin Yang

**Affiliations:** 1 Department of Rehabilitation Medicine Center, Sichuan University West China Hospital, Chengdu, Sichuan, China; 2 Key Laboratory of Rehabilitation Medicine in Sichuan Province, West China Hospital, Sichuan University, Chengdu, China

**Keywords:** autonomic nerve system, chronic non-specific neck pain, conventional physical therapy, eye movement exercise, randomized controlled trial

## Abstract

**Introduction:**

Chronic nonspecific neck pain (CNNP) is a prevalent and disabling musculoskeletal disorder frequently accompanied by cervical sensorimotor deficits and autonomic nervous system (ANS) dysregulation while conventional physical therapy (CPT) can reduce pain and disability, its effects are often limited in autonomic nerve system dysfunction. Eye movement exercise (EME) engages oculomotor–vestibular–proprioceptive networks and stimulates vagal-parasympathetic pathways, offering potential synergistic benefits. However, clinical evidence supporting an EME-integrated rehabilitation regimen in CNNP is scarce. This trial aims to evaluate the efficacy of EME + CPT compared to CPT alone on relieving pain intensity, disability intensity, autonomic regulation, and quality of life for CNNP.

**Methods:**

A single-centre, assessor-blinded randomized controlled trial will recruit 60 adults with CNNP ≥12 weeks. Participants will be randomly assigned (1:1) to either CPT alone or CPT + EME group. Interventions will be administered three times per week for four consecutive weeks (about 60 min per session). The primary outcome is pain intensity (measured using a 100-mm visual analogue scale, VAS). The prespecified primary endpoint is Day 30, and the primary treatment contrast is the between-group difference in mean change in VAS from baseline to Day 30. Secondary outcomes include neck disability index, cervical active range of motion, anxiety and depression severity and health-related quality of life. Heart rate variability (HRV) is prespecified as a secondary mechanistic outcome, whereas oculomotor performance and electrical sensory/pain thresholds, and eye movement accuracy are exploratory outcomes. Data will be collected at baseline, Day 15, and Day 30. The primary analysis will use a linear mixed-effects model under the intention-to-treat principle.

**Discussion:**

This trial will clarify whether integrating structured EME into a standard rehabilitation framework confers incremental short-term clinical benefits over conventional therapy of equal total duration. Exploratory autonomic and oculomotor endpoints will provide insight into underlying physiological associations rather than inferring direct causality, informing future large-scale, long-term rehabilitation trials.

**Clinical Trial Registration:**

https://www.chictr.org.cn/bin/project/edit?pid=293180, identifier ChiCTR2500111782.

## 1 Introduction

Neck pain is a highly prevalent and disabling condition that represents a significant global health burden (1). It is defined as pain localized to the anatomical region of the neck, extending from the superior nuchal line (the base of the skull) to the spinous process of the first thoracic vertebra (T1), and laterally to the margins of the trapezius muscle (1, 2). Chronic non-specific neck pain (CNNP) is defined as neck pain lasting more than 12 weeks without identifiable structural disorders (3). Approximately 50%–85% of adults experience neck pain at least once in their lifetime, and 37% of these symptoms develop into CNNP, often with recurrent episodes within 1–5 years (4). In USA, individuals with CNNP incur mean annual expenditures of $8,512—1.82-fold those of the general populace (5).

Current clinical guidelines recommend exercise and patient education as the primary approaches for managing CNNP (6). However, in routine clinical practice, physiotherapists often focus on correcting postural misalignment and enhancing the function and mobility of key cervical and scapular muscle groups (7). Interventions such as massage, strengthening exercises, stretching, supervised physical therapy, and home exercises can relieve pain and disability, approximately one-quarter of patients still seek additional medical care (8). Current evidence highlights the important role of the autonomic nervous system (ANS) in chronic musculoskeletal pain (9). ANS consists of the parasympathetic (PNS) and sympathetic (SNS) systems. Following local injury, pro-inflammatory mediators excite nociceptors, triggering central reflex arcs that activate both the SNS and the hypothalamic-pituitary-adrenal axis. As the principal parasympathetic conduit, the vagus nerve mediates endogenous analgesia and anti-inflammatory signaling. Recent evidence demonstrates that activation of the cholinergic anti-inflammatory–analgesic pathway attenuates pain, normalizes central sensitization, and improves joint function (10).

Ocular function can influence autonomic nerve function (11). Recent studies suggested that eye movement exercise (EME), especially smooth pursuit exercises, can enhance the function of the brain function (12), then enhance PNS and decrease pain intensity (8, 13). EME stimulates cranial nerves III, IV, and VI-particularly the oculomotor nerve-thereby modulating the oculomotor-vestibular-proprioceptive circuits, central pain-processing pathways, and brainstem autonomic centers to enhance parasympathetic activity, inhibit sympathetic overactivation, and consequently alleviate pain and restore autonomic balance. This modulation may improve pain control, reduce sympathetic overactivation, enhance heart rate variability (HRV), and support emotional regulation (14).

CNNP is frequently accompanied by sensorimotor impairments of the head–eye–neck system, including deficits in cervical proprioception and gaze stability (8, 15). Oculomotor and eye–cervical re-education programs have demonstrated improvements in pain, disability and visual stability (16). Importantly, the oculomotor–vestibular–proprioceptive networks are tightly coupled to brainstem autonomic nuclei (e.g., nucleus tractus solitarius and vagal motor nuclei); modulation of these circuits can therefore influence PNS outflow (17). These lines of evidence support the inclusion of eye-movement training in multimodal rehabilitation for chronic neck pain, both to restore sensorimotor control and to engage brainstem-parasympathetic mechanisms that may attenuate inflammation and chronic pain.

However, the clinical evidence supporting EME in combination with CPT (conventional physical therapy) for CNNP remains limited. This study aims to evaluate the efficacy between EME combine CPT and CPT alone in providing pain relief, improvement of cervical function and quality of life in CNNP.

## 2 Trial status and protocol amendments

This manuscript describes a prospective, single-center, assessor-blinded, two-arm randomized controlled trial conducted at the Rehabilitation Medicine Center, West China Hospital, Sichuan University. Recruitment commenced on 1 December 2025. The trial was designed to enroll 60 participants (30 per group), based on the prespecified sample-size calculation requiring 54 evaluable participants and allowing for an anticipated 10% attrition rate. A total of 62 participants were enrolled, and 54 participants completed the 4-week intervention and all scheduled outcome assessments at the time of the current manuscript revision.

The eligibility criteria, intervention procedures, assessment schedule, and primary outcome were implemented consistently throughout the trial. No substantive changes were made to these trial procedures after recruitment commenced.

During manuscript preparation and peer review, the statistical analysis plan was further specified by replacing the previously described repeated-measures ANOVA/LOCF approach with a longitudinal linear mixed-effects model. This refinement was made to provide a more appropriate framework for analyzing repeated measurements and handling incomplete observations. It did not change the primary outcome, assessment time points, or primary treatment contrast.

## 3 Methods and analysis

### 3.1 Objectives

To compare the effects of two different interventions (EME + CPT vs. CPT) in CNNP patients.

### 3.2 Trial design

This study is designed as a prospective, randomized, controlled, two-arm clinical trial with blinded outcome assessment. Participants will be randomized in a 1:1 ratio to either the EME + CPT group or the CPT only group. Both groups attend supervised treatment sessions three times weekly for four consecutive weeks (12 sessions in total; approximately 60 min per session). The protocol adheres to the SPIRIT guidelines for randomized trial protocols and has been prospectively registered with the Chinese Clinical Trial Registry (18). A flowchart of the trial design is shown in Table 1.

**Table 1:** Flowchart of this study.

Participant timeline: schedule of enrollment, interventions, and assessments.						
	Trial period					
	Enrollment		Post-randomization			Close-out
Timepoint	−t*_1_*	0	t*_1_* (Week 1–2)	t*_2_* (Week 2)	t*_3_* (Week 2–4)	t*_4_* (Week 4)
Enrollment:						
Eligibility screen	X					
Informed consent	X					
Randomization and allocation		X				
Interventions:						
*Intervention group*			X	X	X	X
*Control group*			X	X	X	X
Assessments:						
*Baseline-demographic data*	X					
*Baseline-clinical assessments*		X				
*Mid-term evaluation*				X		
*Final evaluation*						X
*Adverse events*			X	X	X	X

Enrollment (phase duration). Assessmet*_1_*: Eligibility screening, informed consent, and collection of baseline demographic data (e.g., age, gender, BMI, timepoints (0, t*_2_*, t*_4_*): A comprehensive battery of outcome measurements was performed at baseline (0), mid-term evaluation (t*_2_*, Week 2), and close-out (t*_4_*, Week 4). These assessments included: Pain and Disability: Pain Intensity measured by Visual Analog Scale (VAS); Neck Disability Index (NDI). Physiological and Biomechanical Metrics: Heart Rate Variability (HRV) test; Active cervical range of motion (ROM) measured by length; Electrical sensitive/pain threshold; Eye blink and tracking accuracy. Psychological Metrics: Anxiety (SAS) and depression (SDS) scales. Adverse events: Monitored and recorded continuously throughout the 4-week intervention period (t*_1_*–t*_4_*).

### 3.3 Inclusion and exclusion criteria

#### 3.3.1 Inclusion criteria

Eligibility criteria are as follows:

- Men or women aged 18–60 years (19);
- Diagnosed with CNNP (4):
  - Without nerve root symptoms;
  - have chronic nonspecific neck pain lasting ≥12 weeks;
  - have no specific cervical structural pathology;
- VAS score≥30 mm for at least 5 days within 1 week prior to enrollment (20);

#### 3.3.2 Exclusion criteria

Individuals will be excluded from the study for the following reasons:

- a history of cervical spine surgery, significant cervical trauma, cervical fracture, cervical myelopathy, or neurological disorders affecting sensory, motor, or autonomic function (20);
- Severe cardiopulmonary, cardiopulmonary, infectious, or systemic diseases;
- Pregnancy or unstable vital signs;
- medication use that is expected to substantially influence autonomic activity or HRV (21);
- current participation in physiotherapy, manual therapy, acupuncture, exercise therapy, or another treatment specifically targeting neck pain
- Chronic eye disease significntly influence eyesight;
- BMI>30;
- ANS training within the past 6 months;
- Refusal to sign informed consent.

### 3.4 Sample size calculation

The sample size was calculated using PASS 2021 (NCSS, USA)for a two-group repeated-measures design with one between-subject factor (CPT vs. EME + CPT) and one within-subject factor (three time points: baseline, Day 15, Day 30).

The calculation was based on a Geisser-Greenhouse corrected *F* test, assuming a within-subject autocorrelation (*ρ*) of 0.20 across time points and a two-sided significance level (*α*) of 0.05. A standardized effect size index (*σ*_m_/σ = 0.39, with effect standard deviation *σ*_m_ = 0.267 and standard deviation *σ* = 0.683) was estimated from our preliminary pilot dataset (*n* = 5 per group) using the 100-mm VAS primary outcome. Under these parameters, 54 evaluable participants (27 per group) yield 80.5% statistical power to detect the between-group difference and 89.5% power for within-subject changes. Accounting for an anticipated 10% attrition rate (19), the recruitment target was adjusted using *n*’ = *n*/(1–0.10), establishing a final enrollment goal of 60 participants (30 per group).

Because pilot effect sizes derived from very small samples may carry uncertainty and risk overestimation, this estimate was utilized for feasibility-guided planning. A 15-mm between-group difference in VAS change serves as the clinically interpretable threshold based on external chronic neck pain literature (22).

### 3.5 Recruitment strategies

Participants will be recruited at the Rehabilitation Medicine Center of West China Hospital. Recruitment materials are distributed through the rehabilitation centre and social media platforms to broaden outreach. All materials will clearly describe the study's purpose and the voluntary nature of participation.

### 3.6 Randomization and blinding

A computer-generated random allocation sequence was prepared by an independent biostatistician who was not involved in participant recruitment, intervention delivery, or outcome assessment. Allocation concealment was maintained using sequentially numbered, opaque, sealed envelopes stored in an access-controlled administrative location. The corresponding envelope was opened by an independent research assistant only after formal eligibility screening, written informed consent, and completion of baseline assessment. The treating physiotherapist was informed of allocation only after this procedure.

Because both interventions involve active exercise and manual therapy, participants and treating physiotherapists could not be blinded to treatment allocation. Participants were informed that both interventions represented potentially beneficial rehabilitation approaches, without disclosure of the specific study hypothesis or which intervention was expected to be superior.

Outcome assessors and statistical analysts were blinded to treatment allocation. Treating therapists did not participate in outcome assessments. Any accidental assessor unblinding was documented, including the date and circumstances, and reassessment by another blinded assessor was performed where feasible.

### 3.7 Interventions

All participants receive a standardized rehabilitation framework consisting of joint mobility, enhancement, muscle relaxation, muscle strengthening, and cervical motor-control training (6, 23). The seven individual treatment techniques are nested within these four therapeutic components according to their primary therapeutic purpose. This terminology is used consistently throughout the manuscript and Figure 1. All supervised interventions are delivered by physiotherapists with more than 5 years of clinical experience who receive standardized training in the study procedures before participant recruitment.

**Figure 1:**
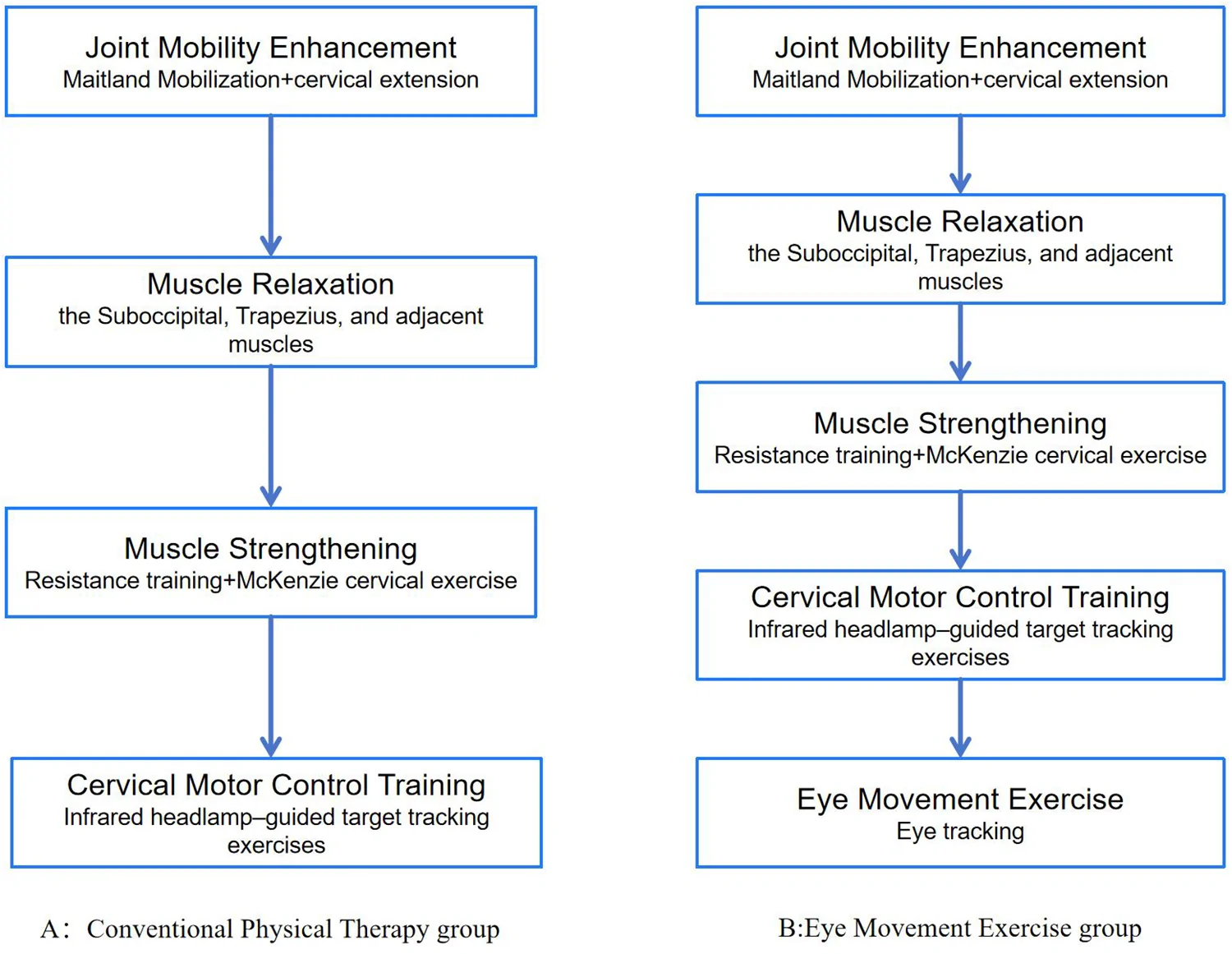
The intervention groups flowchart. **(A)** CPT group; **(B)** EME group.

Treatment is delivered in the same designated rehabilitation room under standardized environmental conditions, including consistent indoor lighting, treatment position, and background visual conditions. Unnecessary visual distractions are minimized. Both groups receive 3 supervised sessions per week for 4 consecutive weeks (23). Attendance, treatment completion, adverse events, and protocol deviations are recorded throughout the intervention period. Participants are instructed not to receive additional interventions specifically targeting neck pain during the study period.

#### 3.7.1 Conventional physical therapy

- Joint mobility enhancement:
  Maitland posteroanterior mobilization is applied from C1 to T12 at an oscillation frequency of approximately 1–2 Hz. Grades I–II are used when pain irritability is the predominant limitation, whereas Grade III-IV is used when restricted mobility is the predominant impairment and the participant can tolerate higher mechanical input (24).
  Cervical extension exercises including chin tucks and sequential segmental extension, are performed for 15 repetitions per set, with two sets per session, using a slow and controlled movement pattern (24)
- Muscle relaxation:
  Manual Muscle release techniques are applied to the suboccipital muscles, upper trapezius, and adjacent cervical muscles. Sustained pressure or stretching is maintained for 30–45s, followed by approximately 10s of rest, and repeated 3–4 times according to participant tolerance (25).
- Muscle strengthening:
  Cervical isometric contractions are performed in four directions: anterior, posterior, left, and right. Participants perform 15 repetitions with a 5-s hold per repetition using a standardized 25-lb (11.3-kg) elastic resistance band (26).
  McKenzie cervical exercise consisting of maximal chin tucks are performed for 15 repetitions with a 5-s hold per repetition (27).
- Cervical motor control training:
  Cervical motor-control training consists of infrared headlamp–guided cervical repositioning exercises. Participants wear an infrared headlamp that projects a visual target onto a wall-mounted target board positioned approximately 2 m in front of them (28, 29). Participants perform controlled cervical repositioning movements while maintaining the projected target within the predefined target area. This component is performed for approximately 5 min per session under physiotherapist supervision.

#### 3.7.2 Eye movement exercise

The eye-movement exercise programme consists of H-shaped tracking (30), Spiral-shaped tracking (30), Near–far convergence training (31). The therapist's right thumb pad serves as the visual target, with a visible target area is approximately 1.5 × 1.5 cm. For pursuit tasks, the target is positioned approximately 40 cm from the participant's eyes. Participants maintain an upright position with the head stabilized in a neutral, stabilized position and follow the target using eye movements while minimizing head movement (8, 30–32).

##### 3.7.2.1 H-shaped tracking

Participants follow the therapist's thumb through the predefined H-shaped trajectory. Two complete repetitions are performed.

##### 3.7.2.2 Spiral tracking

Participants follow the therapist’s thumb through a spiral trajectory consisting of three clockwise and three counterclockwise rotations per set, constituting one complete sequence.

##### 3.7.2.3 Near-far convergence

The target begins at approximately 40 cm from the participant's eyes and is progressively moved toward the participant until the maximum comfortable convergence point is reached, generally within approximately 2.5–6 cm from the nose, and is then returned to approximately 40 cm. This complete sequence constitutes one repetition, and three repetitions are performed (31).

The standardized assessment sequence requires approximately 2 min. The subsequent approximately 5 min of active EME training is individualized according to the participant's observed eye-movement performance, with greater emphasis placed on tasks or directions demonstrating poorer performance. When performance is relatively poor across tasks, the standardized sequence may be repeated during active training. Thus, the total EME duration is approximately 7 min per session.

For H-shaped and spiral tracking, the target is moved at a nominal angular velocity of approximately 10°/s (33). This value serves as a reference rather than an instrumentally verified velocity. Therapists receive standardized training to reproduce the predefined movement trajectories and approximate target velocity. If a participant cannot maintain accurate and smooth pursuit at the nominal velocity, the therapist reduces the target velocity while maintaining the prescribed trajectory.

Corrective lenses are not worn during EME to maintain standardized visual input conditions. All EME sessions are conducted under standardized indoor lighting.

Individualized training emphasis is based on predefined indicators of poorer performance, including reduced smooth-pursuit quality, difficulty maintaining target fixation, inability to complete the prescribed movement excursion, frequent corrective saccades, or reproduction of relevant symptoms (34, 35). Movement speed is progressed only when the participant can maintain accurate target tracking, smooth and continuous pursuit, a stable head position, the prescribed movement excursion, and no clinically relevant symptoms or abnormal ocular responses. Persistent or clinically significant abnormal ocular responses, including suspected nystagmus, are not used as an automatic indication for progression. Such findings prompt clinical reassessment and, where appropriate, referral for further vestibular, neurological, or ophthalmological evaluation.

#### 3.7.3 Treatment duration and between-group comparability

Supervised session duration is standardized at approximately 60 min for both groups to equalize therapist contact time. As in standard multimodal rehabilitation spanning 30–60 min, component durations fluctuate within defined limits according to individual tolerance rather than rigid minute-by-minute schedules.

Manual therapy is delivered within a 15–20-minute window and titrated based on joint responses (decreased hypertonicity, restored accessory glide without guarding, and normalized end-feel without end-range pain). Active exercises span approximately 35–45 min, with intermittent rest intervals scheduled to manage fatigue.

In the EME + CPT group, structured EME (∼7 min) is embedded directly within the 60-minute session, modestly condensing active exercise components and rest buffers (Table 2). This trial evaluates the pragmatic effectiveness of an EME-integrated rehabilitation package versus a time-matched conventional program, rather than an isolated test of EME added to an identical CPT dose.

**Table 2:** Approximate planned time allocation per session across groups.

Intervention		CPT group	EME + CPT group
Joint mobility enhancement	Maitland posteroanterior mobilization	15–20 min	15–20 min
	Cervical extension exercises	3–5 min	3–5 min
Muscle relaxation	Manual Muscle release techniques(the suboccipital muscles, upper trapezius, and adjacent cervical muscles)	10 min	10 min
Muscle strengthening	Cervical isometric contractions	8–10 min	8–10 min
	McKenzie cervical exercise	3–5 min	3–5 min
Cervical motor control training	Cervical motor control training	5 min	5 min
Eye movement exercise	H-shaped tracking, Spiral tracking, Near–far convergence	/	7 min
Rest/transition	Brief rest or transition intervals between treatment components	As clinically indicated	As clinically indicated
Total supervised treatment time		Approximately 60 min/session	Approximately 60 min/session

The predefined approximate duration ranges for the CPT components were the same in both groups. Minor within-range variation was permitted according to participant tolerance and clinical response. In the EME + CPT group, approximately 7 min of EME was incorporated within the overall approximately 60-min supervised treatment session. Brief rest or transition intervals were provided as clinically indicated and were not used as a mechanism to artificially equalize treatment duration.

#### 3.7.4 Home exercise

Both groups receive the same standardized home exercise programme consisting of cervical muscle relaxation, strengthening, and cervical motor-control exercises. Participants are instructed to perform the programme twice daily, with each session lasting approximately 30 min. Adherence is recorded throughout the intervention period.

#### 3.7.5 Treatment fidelity

Treatment fidelity is monitored through: standardized therapist training before recruitment; use of the predefined intervention protocol; recording of attendance and treatment completion; documentation of protocol deviations; documentation of adverse events; recording of EME task selection and training emphasis; and standardized treatment environment.

#### 3.7.6 Safety monitoring and stopping criteria

Adverse events (AEs) are monitored from the first intervention session through the Day 30 assessment. AEs are documented by the treating physiotherapist or clinical coordinator and evaluated for severity, relatedness to the intervention, and expectedness.

AEs are classified as mild, moderate, or severe according to their clinical impact. Mild AEs are transient symptoms that do not require additional medical intervention or result in substantial functional limitation. Moderate AEs are symptoms requiring clinical management, temporary interruption of treatment, or additional observation. Severe AEs are events requiring urgent medical evaluation, hospitalization, or resulting in substantial clinical deterioration.

Causality is classified as unrelated, possibly related, probably related, or definitely related to the intervention. Expected intervention-related events include transient neck soreness, fatigue, mild dizziness, ocular discomfort, or transient visual instability.

EME is immediately interrupted if participants develop clinically relevant dizziness, nausea, headache, blurred vision, diplopia, significant eye discomfort, suspected nystagmus, marked deterioration in smooth pursuit, or other clinically relevant symptoms. Participants are observed and managed according to clinical need, including up to 30 min of supervised rest when appropriate.

Serious or unresolved adverse events are referred for appropriate clinical management and reported to the institutional ethics committee in accordance with applicable requirements. Study-related AEs are followed until resolution or clinical stabilization where feasible. Participants may discontinue the intervention for safety reasons, participant request, clinically significant deterioration, or other circumstances judged by the investigator to make continued participation inappropriate.

### 3.8 Outcome measures

Assessments will be conducted at baseline, day 15 and day 30. Day 30 is the prespecified primary endpoint. The primary treatment contrast is: between-group difference in mean change in VAS from baseline to Day 30.The Day 15 assessment characterizes the intermediate trajectory of treatment response and does not replace the Day 30 primary endpoint. No post-treatment follow-up beyond Day 30 is included. Therefore, this trial evaluates short-term efficacy rather than persistence of treatment effects. Outcome assessors remain blinded to treatment allocation.

#### 3.8.1 Primary outcome measures

##### 3.8.1.1 Visual analog scale (VAS) score

Neck pain intensity is measured using a 100-mm horizontal VAS (36). 0 mm = no pain;100 mm = worst imaginable pain. Participants rate their average neck pain during the preceding 7 days. VAS is measured at baseline, Day 15, and Day 30. The primary efficacy contrast is the between-group difference in mean change from baseline to Day 30. A 15-mm between-group difference in change on the 100-mm VAS is considered a clinically interpretable difference based on previous evidence in chronic nonspecific neck pain.

#### 3.8.2 Secondary outcome measures

##### 3.8.2.1 Neck disability index

NDI measures neck disability. The NDI is a self-report instrument to assess the degree of disability in patients with neck pain, which includes pain, person care, weight pain, reading, headache, concentration, work, driving, sleep and leisure. Each item has 0–5 degree to describe the influence of the neck pain related symptoms, which 0 means “painless” and 5 means “the worst pain imaginable” (NDI = total score of each item/number of items completed by the subject ×5 × 100%). Joshua A. et al. confirmed that NDI has excellent reliability and validity (37).

##### 3.8.2.2 Active cervical range of motion (AROM) measured by length

Cervical AROM is assessed in six directions using a flexible tape measure (6). Participants are seated in the same standardized position at each assessment. They perform each movement actively to the maximum comfortable range while avoiding compensatory trunk movement. Measurements include: flexion; extension; left rotation; right rotation; left lateral flexion; and right lateral flexion.

Flexion and extension are quantified as the linear distance between the chin and sternal notch. Rotation is measured as the linear distance between the chin tip and the contralateral shoulder landmark. Lateral flexion is measured as the distance between the ipsilateral tragus and shoulder tip.

Each movement is measured three times, and the mean value is used for analysis. The same assessor, participant positioning, anatomical landmarks, and measurement procedure are maintained across all assessment time points. Linear-distance measurement is used because it is a practical clinical method for repeated cervical ROM assessment and has demonstrated acceptable reliability (38).

##### 3.8.2.3 Anxiety (SAS) and depression (SDS) scale

According to the frequency of symptoms in the past week, subjects rated 20 items on a scale of 1–4 (1 = none/rarely, 4 = most of the time). The calculation method was to sum the scores of each item (depression needed to reverse score 10 items) to calculate the original score, and then the original total score × 1.25 (rounded) to obtain the standard score. Pang et al. and Steven et al. confirmed the good reliability and validity of SAS and SDS (39).

##### 3.8.2.4 Quality of life

Health-related quality of life will be measured using the World Health Organization Quality of Life Brief Version (WHOQOL-BREF) (40). The WHO QOL-BREF has demonstrated good cross-cultural validity and reliability, with Cronbach’s alpha values typically exceeding 0.7 for all domains in various patient populations, including those undergoing rehabilitation (40).

##### 3.8.2.5 Secondary mechanistic outcome: HRV

HRV is assessed using a CM700 heart rate variability analyzer (Sichuan Credit Pharmaceutical Co., Ltd., China). Participants rest quietly in a standardized seated position for 5 min before recording and breathe spontaneously without deliberate respiratory pacing. Participants are instructed to abstain from caffeine, tea, alcohol, and vigorous physical activity for at least 24 h before assessment and to abstain from nicotine for at least 24 h before assessment (41, 42). Medications expected to substantially influence autonomic activity are avoided when clinically appropriate, and participants are screened for clinically significant arrhythmia.

A continuous 5-min ECG recording is obtained at a sampling frequency of 500 Hz under spontaneous breathing. Repeated HRV assessments are scheduled at approximately similar times for each participant when feasible; in practice, assessments within the same participant generally occur within a 2-h window, and the exact assessment time is recorded. Potential circadian variation is acknowledged as a methodological limitation.

Raw inter-beat interval data are exported and independently processed in R using the RHRV package. Artifact filtering and ectopic-beat removal are performed before calculation of prespecified HRV indices, including SDNN, RMSSD, pNN50, LF, HF, LF/HF ratio, and SD1.

The CM-series device was used for ECG acquisition and recording. We did not identify an independent published validation study specifically evaluating the validity or reliability of this device; therefore, no device-specific validity or reliability claim is made.

##### 3.8.2.6 Electrical sensitive/pain threshold (43)

Sensory and pain thresholds are assessed using a PainVision PS-2100 system. Participants rest for 5 min before assessment and remain seated in a standardized posture: feet shoulder-width apart; knees flexed at approximately 90°; hands resting on the knees; and eyes closed.

The sensory threshold test is performed over 100 s and the pain threshold test over 20 s, with a current limit of 128 μA and voltage limit of 200 V. Three consecutive measurements are obtained with a 1-min rest interval between trials. The mean of the three measurements is used for analysis.

##### 3.8.2.7 The eye blink and tracking accuracy

Oculomotor performance is assessed under standardized visual target and head-position conditions consistent with the EME procedures. The assessor records blink frequency (44), successful target tracking, loss of fixation, failure to complete the prescribed movement path, corrective eye movements, and other predefined abnormal ocular responses.

Successful tracking is defined as maintaining gaze on the target throughout the prescribed trajectory without loss of fixation, voluntary head movement, or failure to complete the required movement path.

## 4 Statistical analysis

Statistical analyses will be performed using RStudio version 2024.12.0 and IBM SPSS Statistics version 27. All statistical tests will be two-sided with *α* = 0.05. Continuous variables will be summarized as mean ± standard deviation or median (interquartile range), as appropriate. Categorical variables will be summarized using frequencies and percentages.

### 4.1 Primary analysis and longitudinal analysis

The primary analysis will follow the intention-to-treat (ITT) principle, analyzing all randomized participants according to their allocated treatment arm. The primary outcome will be analyzed using a constrained longitudinal linear mixed-effects model estimated via restricted maximum likelihood (REML). To avoid analytical redundancy between baseline as an outcome time point and baseline as an adjusting covariate, post-baseline measurements (Day 15, Day 30 VAS) will serve as the repeated dependent variations. Fixed effects include treatment group (CPT vs. EME + CPT), assessment time point (day 15, day 30), and the treatment-by-time interaction, with baseline VAS score included as a continuous fixed covariate. Participant identity will be modeled as a random intercept to account for repeated within-subject correlation. The primary treatment effect will be evaluated as the model-adjusted between-group difference in mean change from baseline to Day 30, reported alongside its 95% confidence interval and two-sided *p*-value. The treatment-by-time interaction will characterize the trajectory of clinical response across post-baseline intervals. The observed treatment effect will be interpreted in reference to the prespecified 15-mm MCID threshold.

### 4.2 Secondary outcomes

Key secondary clinical outcomes will be analyzed using analogous mixed-effects models where appropriate. HRV will be analyzed as a secondary mechanistic outcome. Oculomotor performance and electrical sensory/pain thresholds will be analyzed as exploratory outcomes. Effect estimates and 95% confidence intervals will be reported.

### 4.3 Prespecified covariate-adjusted sensitivity analyses

The robustness of the primary treatment effect to clinically relevant baseline characteristics will be examined using prespecified sensitivity analyses. These analyses will additionally adjust for baseline age, and NDI score. Because psychological symptoms may influence pain perception and treatment response, the potential influence of baseline anxiety and depressive symptoms will also be examined in separate sensitivity models by adding baseline SAS or SDS score, respectively. SAS and SDS will not be entered simultaneously into the same model because of their potential correlation and the relatively small sample size. Covariates will be selected on the basis of clinical relevance and prespecified methodological considerations rather than their statistical significance in the observed data. BMI will not be included as a prespecified covariate.

### 4.4 Missing data

The primary LMM will use all available repeated outcome observations under a missing-at-random (MAR) assumption. The number, timing, and reasons for missing outcome data will be documented and summarized by treatment group. Missingness will be described as intermittent missingness or monotone dropout. For participants with intermittent missing observations, all available measurements will be included in the LMM without imputing individual missing observations. For participants who discontinue before Day 30, all available outcome measurements will be retained in the primary analysis.

The robustness of the primary findings to different assumptions regarding missing data will be evaluated using prespecified sensitivity analyses. A complete-case analysis will be conducted as a first sensitivity analysis. Multiple imputation under the MAR assumption will be used as an additional sensitivity analysis when primary outcome data are missing. The imputation model will include treatment group, baseline VAS, available longitudinal VAS measurements, age, sex, and baseline NDI. A delta-adjusted multiple-imputation analysis will additionally be performed to examine the potential impact of departures from the MAR assumption on the estimated treatment effect.

A per-protocol analysis will also be conducted as a supportive sensitivity analysis. The per-protocol population will consist of participants who meet the prespecified adherence criterion of completing at least 80% of the scheduled supervised treatment sessions.

All prespecified covariates, missing-data procedures, and sensitivity analyses will be defined before database lock and before examination of unblinded treatment-effect estimates. Any analyses subsequently undertaken in response to unexpected findings will be explicitly identified as exploratory or *post hoc*.

### 4.5 Per-protocol analysis

The per-protocol population will include participants who complete at least 80% of scheduled intervention sessions without major protocol deviations. Per-protocol analyses will be considered supportive sensitivity analyses rather than replacements for the primary ITT analysis.

### 4.6 Non-normal outcomes

When model assumptions are substantially violated and cannot be adequately addressed through appropriate transformation or model specification, generalized linear mixed-effects models or other appropriate prespecified alternatives will be considered.

### 4.7 Multiplicity

Because the study has one prespecified primary outcome, the primary hypothesis will not be divided across multiple primary endpoints. Bonferroni-adjusted contrasts will be used where multiple post-baseline pairwise comparisons are performed. Exploratory outcomes will be explicitly interpreted as hypothesis-generating.

### 4.8 Exploratory regression

Exploratory regression analyses may be conducted to examine clinically plausible associations between HRV measures and pain or disability outcomes. Such analyses will be clearly labeled as exploratory and will not be interpreted as evidence of causal mediation.

## 5 Discusion

Chronic nonspecific neck pain is a multifactorial condition involving interactions among pain processing, cervical sensorimotor function, psychological factors, and potentially autonomic regulation. Conventional rehabilitation remains an important component of management, but persistent symptoms and sensorimotor impairments may warrant consideration of complementary rehabilitation strategies. Previous studies have identified interactions between cervical and oculomotor systems in individuals with neck pain, and eye–cervical re-education approaches have shown potential effects on pain, disability, and sensorimotor function. The present trial will evaluate whether incorporating structured EME into standardized conventional physical therapy provides additional short-term clinical benefit.

The proposed relationships among eye movement, sensorimotor integration, autonomic modulation, and pain remain exploratory. HRV is therefore included as a secondary mechanistic outcome to investigate whether changes in autonomic modulation accompany clinical changes. HRV will be interpreted as an indirect physiological marker rather than a direct measurement of vagal activity or autonomic balance (9). Similarly, associations among changes in pain, HRV, and oculomotor performance will not be interpreted as evidence of causal mediation.

### 5.1 Strengths

This study has several methodological strengths. First, it uses a randomized controlled design with concealed allocation and blinded outcome assessment. Second, both groups receive approximately 60 min of supervised rehabilitation per session, with predefined duration ranges for the major CPT components. Third, the CPT and EME procedures are operationally standardized, including intervention dosage, target characteristics, progression criteria, safety monitoring, and treatment-fidelity procedures. Fourth, the study clearly distinguishes the primary clinical outcome from secondary mechanistic and exploratory outcomes within a prespecified longitudinal statistical framework. Finally, the trial incorporates both clinical and physiological assessments while explicitly limiting mechanistic interpretation to exploratory associations.

### 5.2 Limitations

Several limitations should be considered. First, participant and therapist blinding is not feasible because of the nature of the interventions. Although participants were informed that both interventions could be beneficial and outcome assessors and statistical analysts remained blinded, expectation and performance bias cannot be completely excluded.

Second, although the predefined approximate duration ranges of the individual CPT components were the same in both groups, minor within-range variation could occur according to participant tolerance and clinical response. In addition, the EME + CPT group incorporated approximately 7 min of EME within the overall supervised treatment session. The study therefore evaluates the clinical effectiveness of an EME-integrated rehabilitation programme rather than the isolated physiological effect of EME.

Third, the sample-size calculation was based on a standardized effect estimate derived from a small pilot sample of five participants per group. Although 54 evaluable participants were required under the prespecified PASS assumptions and the planned total sample size was set at 60 after allowing for 10% attrition, the pilot-derived effect estimate remains uncertain and may overestimate the true treatment effect. Therefore, the statistical power of the study may be lower than anticipated if the true effect is smaller than the pilot estimate. The findings should consequently be interpreted with attention to the estimated treatment effect, its 95% confidence interval, and clinical relevance.

Fourth, no follow-up beyond Day 30 is included. Consequently, the study evaluates short-term treatment effects and cannot determine whether any observed benefits persist over longer periods.

Fifth, oculomotor performance is assessed manually rather than using instrument-based eye tracking. Although standardized training and predefined criteria are used to reduce observer variability, manual assessment may have lower measurement precision. These outcomes are therefore considered exploratory.

Future studies should include larger samples, longer follow-up, and more objective assessment of oculomotor performance. Where the isolated effect of EME is of interest, time- and attention-matched control conditions should also be considered.
