# Supplementary material for: Effects of eye movement exercise combined with conventional physical therapy on chronic nonspecific neck pain: a randomized controlled trial protocol
Source: Front Sports Act Living. 2026 Oct 14;8:1929676. doi: 10.3389/fspor.2026.1929676 (PMC13713468; doi:10.3389/fspor.2026.1929676)
Supplement: Supplementary file 1 [file Supplementaryfile1.docx]

Supplementary Material

# 1 Supplementary Data

Ethical approval

| Department (Specialty): Department of Rehabilitation Medicine / Faculty of Rehabilitation Sciences |

| Principal Investigator & Title: Yang Lin / Senior Technologist |

| Project Title: Effects of Eye Movement Training Combined with Conventional Physical Therapy versus Conventional Physical Therapy Alone on Chronic Non-Specific Neck Pain: A Randomized Clinical Trial |

| Review Mode: ■ Committee Review □ Expedited Review |

| List of Reviewed Documents: See Attachment |

| Meeting Date/Location: 2025-10-21 / Administrative Building, 10th Floor Conference Room |

Review Opinion

The qualifications of the researcher comply with ethical requirements.

The research protocol and informed consent form generally meet ethical requirements.

Review Result

■ Approved

□ Approved with Modifications

□ Re-review Required

□ Not Approved

□ Suspension or Termination of Study

Frequency of Continuing Review

□ 3 months □ 6 months ■ 1 year □ N/A

Notes

Please adhere to relevant Chinese laws and regulations (Measures for Ethical Review of Life Science and Medical Research Involving Human Subjects, Measures for Ethical Review of Biomedical Research Involving Human Subjects, etc.), as well as the WMA Declaration of Helsinki and the CIOMS International Ethical Guidelines for Health-related Research Involving Humans. Conduct the clinical trial (research) in accordance with the protocol and informed consent form approved by the Ethics Committee, and protect the rights and interests of research participants.

All activities involving human genetic resources in our hospital must strictly comply with the Regulations of the People's Republic of China on the Management of Human Genetic Resources and relevant service guidelines, and undergo administrative approval for human genetic resources. After approval by our Ethics Committee, researchers must apply for administrative approval on the website of the Department of Science and Technology of the Ministry of Science and Technology (Consultation Hotline: 028-85422851).

During the study, any modifications to the research protocol, informed consent form, recruitment advertisements, or other materials provided to research participants should be submitted by the applicant via an amendment application.

In the event of a serious adverse event, the applicant must promptly submit a Serious Adverse Event Report; following an emergency report, a detailed follow-up report of the serious adverse event should be submitted as soon as possible.

If any situation occurs that may significantly affect the conduct of the trial (research) or increase the risk to research participants, the applicant must promptly submit a written report to the Ethics Committee.

If participants who do not meet the inclusion criteria or meet the exclusion criteria are enrolled, or if participants who meet the discontinuation criteria are not withdrawn, or if incorrect treatment or dosage is administered, or if prohibited concomitant medications are given, or if any other deviations from the protocol that may adversely affect participants' rights/health or the scientific integrity of the study occur, the sponsor/monitor/investigator must submit a Protocol Deviation Report.

If the applicant suspends or terminates the clinical trial (research) early, a Suspension/Termination Report must be submitted in a timely manner.

Upon completion of the clinical trial (research), the applicant must submit a Closure Report.

Clinical research may not proceed without ethical approval.

This approval is valid for one year. If the study is not initiated within one year, this approval will automatically expire. If initiated within one year, this approval remains valid long-term. Please submit annual and periodic follow-up review reports in a timely manner.

According to the requirements of the International Committee of Medical Journal Editors (ICMJE), all clinical research involving human subjects or human specimens must be registered. Researchers receiving this approval are requested to register with the Chinese Clinical Trial Registry before the start of the clinical study. Please use our institution's public account (send an email to the Clinical Research Management Department at hx@xuexi.com to apply; Contact: 028-85422851) and log in to the following website for clinical research registration: http://www.chictr.org.cn. The unique registration number generated upon successful registration must be sent promptly via email to the Ethics Committee Office (ethics@wchscu.cn), as it is a mandatory item for ethical follow-up review.

Signature:

Chairperson (Signed and Sealed):

Date: October 24, 2025

Attachment: Documents Reviewed by the Ethics Committee

Initial Review Application Form

Research Protocol (Version No.: V3.0; Version Date: 2025-09-17)

Informed Consent Form (Version No.: V2.0; Version Date: 2025-09-17)

Curriculum Vitae of Principal Investigator

Proof of Funding Source

Recruitment Advertisement

# 2 Supplementary Figures and Tables

Figure. 1 Flowchart of this study

Figure 2. The intervention groups flowchart,A:CPT group; B:EME group.
